# Regulation of oncogenic PI3-kinase signaling by JARID1B

**DOI:** 10.18632/oncotarget.14790

**Published:** 2017-01-21

**Authors:** Nicole D. Facompre, Kayla H. Harmeyer, Devraj Basu

**Affiliations:** Department of Otorhinolaryngology-Head and Neck Surgery, The University of Pennsylvania, The Wistar Institute and VA Medical Center, Philadelphia, PA, USA

**Keywords:** oral cancer, cancer stem cells, squamous cell carcinoma, JARID1B, PI3K

The central role of PI3K/Akt signaling in survival, anabolic metabolism, and division of normal cells is exploited in multiple cancers including oral squamous cell carcinomas (OSCCs), where it is the most mutated oncogenic pathway. Despite development of potent inhibitors of key nodes in the pathway, these drugs have usually shown only modest efficacy against cancer. Our recent detection of a non-dividing cell state within OSCCs that survives anti-PI3K drugs offers new insight into how intra-tumor heterogeneity supports innate resistance to PI3K targeting [1]. Not meeting strict criteria for full cell cycle exit, this “G_0_-like” cell fraction was previously shown to arise stochastically during asymmetric division via degradation of Akt [2]. G_0_-like cells were further defined by molecular hallmarks of low reactive oxygen species and total RNA levels as well as high p27^kip1^ and Hes1. In our study, they demonstrated enhanced clonal sphere and xenograft formation capacity despite lacking the conventional markers of oral cancer stem cells. Marked expansion of the G_0_-like fraction by PI3K inhibitors allowed it to limit dependence on PI3K/Akt signaling by creating a quiescent reservoir that retains high malignant potential.

We further demonstrated that G0-like OSCC cells are primed to exit quiescence by upregulation of the histone demethylase JARID1B, which provides a mechanism for their enhanced malignant potential. JARID1B has shown diverse, context specific roles in epigenetic regulation of cell phenotype in normal development and multiple cancer types. At least some oncogenic functions of JARID1B are mediated by its demethylase activity repressing transcription at select promoters by removing the H3K4me3 mark. JARID1B’s association with stem cell-like traits in malignancy was first shown in melanoma [3] prior to our related observations made in OSCC [1]. Both cancers contain a cell fraction where high JARID1B levels mediate efficient clonal sphere formation and *in vivo* tumorigenicity. JARID1B upregulation in G_0_-like cells promoted hyper-activation of Akt signals as well as cell cycle progression, which led to slow-cycling self-renewal rather than rapid division [1]. In contrast to the G_0_-like state, these JARID1B^high^ OSCC cells displayed oral cancer stem cell markers and enhanced expression of EMT-related transcripts. They were further distinguished by high sensitivity to PI3K inhibitors [1] despite resisting other cytotoxic and targeted agents [3]. Remarkably, stable silencing of JARID1B expanded the G_0_-like fraction while simultaneously attenuating its tumorigenicity. Together, these observations support a model in which G_0_- like cells are poised for JARID1B-mediated transition to a slow-cycling, stem cell-like state with hyperactive PI3K/ Akt signaling and PI3K inhibitor sensitivity.

**Figure 1:**
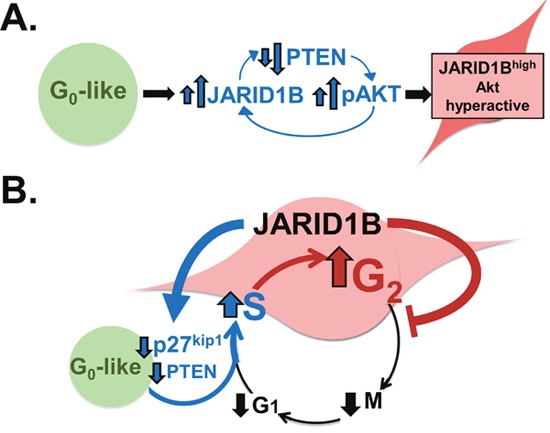
Working models of JARID1B-mediated regulation of Akt activation and cell cycle progression **A.** Akt activation increases JARID1B levels, which initiates a feed-forward loop of further Akt activation by repressing PTEN. **B.** JARID1B expands the G_2_ phase of the cell cycle profile and creates a slow-cycling state by promoting G_1_-S transition while delaying G_2_ transit.

The interactions between JARID1B expression and Akt activation that create a stem cell-like phenotype remain to be fully defined. In hepatocellular carcinoma, JARID1B was previously shown to repress the PTEN promoter directly [4] and thus may enhance PI3K/Akt signaling by this mechanism. However, JARID1B levels also are regulated downstream of Akt based on our observation that ectopic Akt activation increases JARID1B transcript and protein levels [1]. Combining this effect with JARID1B-mediated PTEN repression is predicted to amplify Akt activation in a feed-forward loop (Figure 1A). JARID1B may also promote G_1_-S progression in G_0_- like cells based on evidence for it repressing the p27^kip1^ promoter [5]. Interestingly, the resulting JARID1B^high^ state has a cell cycle profile biased toward G_2_/M, creating an appearance of G_2_/M checkpoint activation. In the absence of genotoxic stress, this finding may be related to an observation in pluripotency, where actions that prolong S/ G_2_ but not G_1_ favor stem cell maintenance and may serve to maximize germline genome integrity [6]. Slowed transit through G_2_ in JARID1B^high^ cells might similarly protect this stem cell-like state from cancer genome instability and is consistent with their resistance to DNA damaging drugs [3]. By these mechanisms, JARID1B-mediated cell cycle regulation may stabilize a slow cycling population (Figure 1B) whose mesenchymal-like traits subsequently facilitate aspects of invasion and metastasis.

These findings integrate known mechanisms of PI3K inhibitor resistance into a conceptual framework where PI3K signal dependence is regulated by flux among distinct chromatin states. Although PI3K/Akt is a major oncogenic driver, its activation appears remarkably heterogeneous and plastic in any single tumor. As a result, compensation to PI3K targeting occurs not only at the level of signaling networks in individual cells but also through dynamics among multiple cell states cooperatively sustaining cancer progression. Based on this concept, G_0_- like cells might provide the locus for the rapid MAPK pathway compensation that is known to occur under PI3K inhibition. The toxicity of combining PI3K and MAPK pathway inhibition underscores the potential utility of alternate strategies to address the epigenetic plasticity that allows cancers to escape inhibition of proliferative signaling. In this regard, a potent and specific new generation of JARID1 family inhibitors is an alternative to PI3K inhibition that could address JARID1B-mediated amplification of PI3K signals [7]. Specifically, targeting JARID1B holds promise to both deplete Akt-hyperactive states and allow combined inhibition of MAPK signals or other targets with acceptable toxicity.
